# Neural correlates of pure apathy in multiple sclerosis: An exploratory neuroimaging study

**DOI:** 10.1111/jnp.70041

**Published:** 2026-03-19

**Authors:** Valentina Torchia, Vera Gramigna, Mariachiara Gaita, Daniele Spitaleri, Antonio Costanzo, Liana Palermo, Florindo d'Onofrio, Simona Raimo

**Affiliations:** ^1^ Department of Health Sciences “Magna Graecia” University of Catanzaro Catanzaro Italy; ^2^ Department of Medical and Surgical Sciences “Magna Graecia” University of Catanzaro Catanzaro Italy; ^3^ Department of Psychology University of Campania “Luigi Vanvitelli” Caserta Italy; ^4^ Neurology Unit San Giuseppe Moscati Hospital Avellino Italy; ^5^ Department of Humanities University of Federico II Naples Italy

**Keywords:** apathy, basal ganglia, depression, MRI, multiple sclerosis

## Abstract

Pure apathy is relatively common in people with Multiple Sclerosis (PwMS), but its neural mechanisms remain unexplored. Therefore, we investigated the neural correlates of pure apathy in PwMS, differentiating it from depression. Sixty‐two PwMS underwent neuropsychological assessments and Magnetic Resonance Imaging scans. Pure apathy is primarily associated with lesions in the cortical‐limbic‐subcortical system implicated in goal‐directed behaviour and motivation. Apathy and depression arise from distinct yet partially overlapping neural mechanisms, with basal ganglia and limbic structures playing a key role.

## INTRODUCTION

Apathy is characterized by a lack of motivation, interest and emotional responsiveness, independent of disturbances in consciousness, cognitive impairment or emotional distress. It manifests as impaired goal‐directed behaviour, reduced productivity and social engagement. Apathy has been widely recognized across various neurological and psychiatric disorders, including multiple sclerosis (MS), but it remains overlooked in clinical practice, despite its association with lower quality of life, greater physical disability and cognitive impairment in individuals with MS (Raimo et al., 2016, 2020). One of the main challenges is to distinguish apathy from depression, particularly when the two conditions coexist. While they share overlapping symptoms, their differentiation is crucial for appropriate intervention and prognosis, as they present distinct risk factors for disease progression. Despite its clinical relevance, the neural correlates differentiating apathy and depression in MS remain poorly understood. Different studies have examined the neurobiological mechanisms underlying apathy, identifying focal damage in regions such as the medial frontal cortex, anterior cingulate cortex, inferior frontal gyrus, parietal cortex, caudate nucleus, putamen, ventral tegmental area and ventral pallidum (Raimo et al., 2019). However, these studies often fail to distinguish apathy from depression, limiting our understanding of their distinct neural substrates. To date, only two studies have specifically investigated the neural correlates of apathy in MS. Cote et al. (2023) found that older adults with MS with higher levels of apathy had reduced caudate volumes, suggesting that such structures, particularly vulnerable to MS‐related damage, may play a role in apathy severity. Tazza et al. (2024), using diffusion tensor imaging (DTI), identified microstructural alterations in frontal and prefrontal cortical regions, as well as in the caudate nucleus and fronto‐striatal white matter pathways, reinforcing the involvement of fronto‐striatal circuits in MS‐related apathy. However, neither study differentiated cases of pure apathy, that is, apathy occurring independently of depression, leaving unresolved the question of whether specific lesion patterns uniquely characterize apathy in the absence of affective symptoms.

Based on this evidence, the present study aimed to fill this gap by examining the neural correlates of pure apathy in MS. Specifically, we sought to identify whether distinct lesion patterns differentiate pure apathy, pure depression and their co‐occurrence. Grounded in previous literature (Cote et al., 2023; Raimo et al., 2019), we hypothesized that pure apathy would be primarily associated with lesions in fronto‐striatal circuits involved in motivation and goal‐directed behaviour, whereas depression would be associated with cortico‐limbic circuits. By testing these hypotheses, our objective was to clarify whether motivational and affective symptoms in MS are supported by dissociable neural substrates. Moreover, the study aims to refine the neurobiological understanding of apathy in MS, improve the differential diagnosis between apathy and depression and provide a structural basis for developing targeted neuropsychological and rehabilitative interventions for people with MS.

## MATERIALS AND METHODS

### Participants

Sixty‐two PwMS (15 males and 47 females) with relapsing‐remitting clinical course were recruited from the Multiple Sclerosis Centre of Moscati Hospital in Avellino (Italy) and underwent neuropsychological assessment and Magnetic Resonance Imaging scans. They were classified into four distinct groups, based on clinical criteria and validated cut‐off scores for clinically significant apathy (Apathy Evaluation Scale, Self‐report version, AES‐S ≥36/72) and depression (Hamilton Depression Rating Scale, HDRS‐17 ≥15/54): PwMS without apathy or depression (A−D−), PwMS with pure apathy without depression (A+D−), PwMS with depression only (A−D+) and PwMS with both apathy and depression (A+D+). Information on pre‐morbid affective and personality traits was obtained during the clinical interview. No participant disclosed a history of major depression or persistent apathy prior to MS onset, ensuring that the affective symptoms investigated reflected MS‐related manifestations rather than pre‐existing traits. The detailed description of the neuropsychological assessment, neuroimaging acquisition, lesion identification and statistical analyses is reported in Supplementary Material S1.

## RESULTS

### Demographic and clinical characteristics

The demographic and clinical characteristics of each group are shown in Table 1. The Kruskal–Wallis test showed a significant group effect on EDSS (*p* = .013) and MMSE (*p* = .013). Post‐hoc analyses showed that A+D+ had significantly lower MMSE scores than A−D−, and that PwMS with apathy (A+D− and A+D+) had higher disability levels than PwMS without apathy (A−D− and A−D+).

**TABLE 1:** Demographic and clinical variables (mean ± SD) of the four subgroups of patients with Multiple Sclerosis: (A−D−) without apathy/depression, (A+D−) ‘pure apathy’/no depression, (A−D+) depression and (A+D+) both apathy and depression. For each variable summary statistics from Kruskal–Wallis (*H*)/Chi‐square (χ^2^) analysis are reported.

	A−D− (*n* = 20)	A+D− (*n* = 12)	A−D+ (*n* = 14)	A+D+ (*n* = 16)	*H*/*χ* ^2^	*p*
Demographic variables						
Age (years)	40.45 ± 10.32	49.75 ± 16.02	42.79 ± 8.47	48.69 ± 8.32	7.659	.054
Education (years)	13.05 ± 3.56	10.50 ± 4.23	12.36 ± 3.54	11.88 ± 3.42	3.289	.349
Sex (F/M)	15/5	6/6	12/2	14/2	6.307	.098
Clinical variables						
EDSS	2.85 ± 1.51	4.33 ± 1.74^a^	2.96 ± 0.66^b^	4.30 ± 1.78^a^	10.794	.013
Duration of disease (months)	114.00 ± 65.78	151.25 ± 121.50	90.00 ± 59.68	141.75 ± 76.80	4.717	.194
Age at onset of disease (years)	32.10 ± 10.74	38.17 ± 14.01	35.36 ± 8.61	35.44 ± 7.83	2.236	.525
Global cognitive functioning						
MMSE	27.46 ± 1.08	25.09 ± 3.56	26.68 ± 1.33	24.71 ± 3.60^a^	10.744	.013
Behavioural assessment						
AES‐S	25.35 ± 3.99	38.92 ± 5.98^a^	26.57 ± 4.62^b^	40.25 ± 9.45^a^, ^c^	34.019	<.001
HDRS	7.70 ± 3.96	10.92 ± 2.91	16.00 ± 5.04^a^, ^b^	18.75 ± 6.40^a^, ^b^	33.928	<.001

Abbreviations: AES‐S, Apathy Evaluation Scale; EDSS, Expanded Disability Status Scale; F, female participants; HDRS, Hamilton Depression Rating Scale; M, male participants; MMSE, Mini‐Mental State Examination.

^a^ Significantly different from A−D− at *p* < .05.

^b^ Significantly different from A+D− at *p* < .05.

^c^ Significantly different from A−D+ at *p* < .05.

### Lesion‐symptom mapping

**FIGURE 1:**
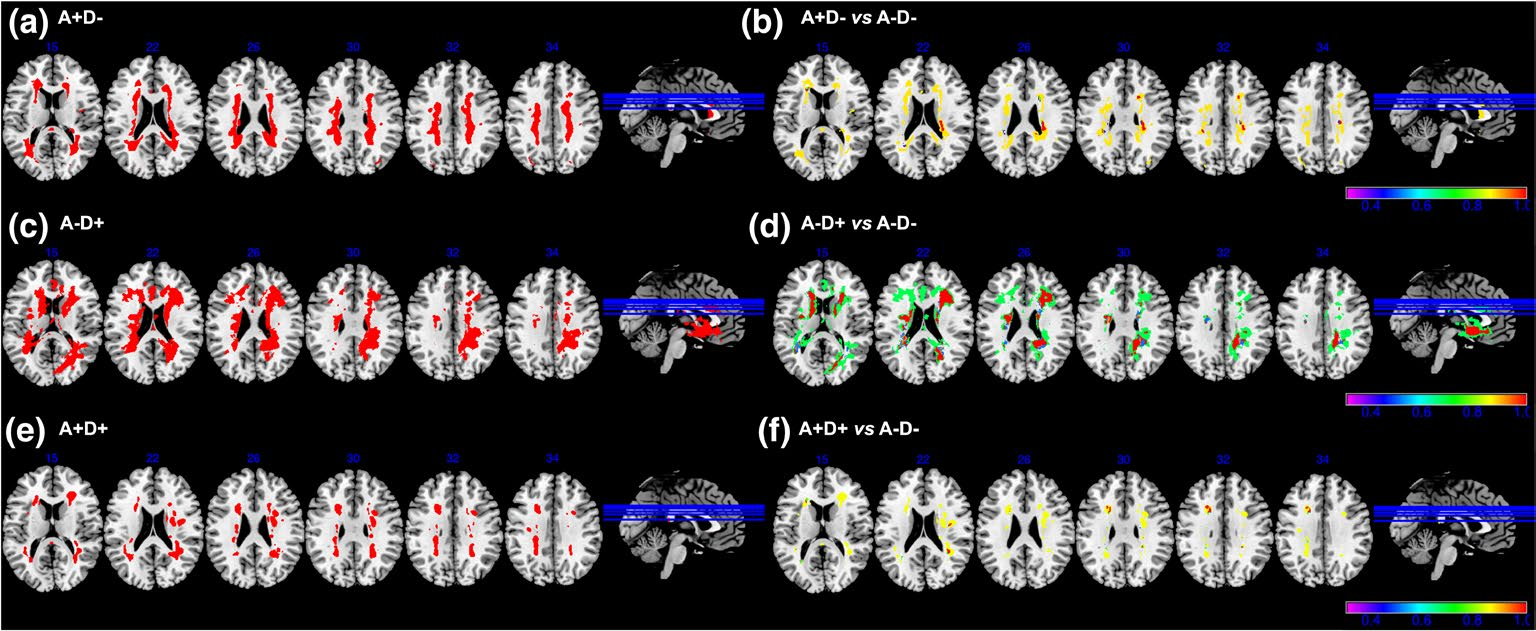
Lesion‐symptom mapping for the PwMS groups. Multislice maps of overlapping results superimposed on axial slices of a standard brain from a single normal subject (MRIcron: ch2.betnii.gz) for (a) A+D−; (c) A−D+; (e) A+D+. Subtraction analysis results for (b) A+D− versus A−D−; (d) A−D+ versus A−D−; (f) A+D+ versus A−D− comparisons. The colour map indicates the frequency of pathology‐related lesions (maximal: Red blobs).

In the A+D− group, lesion overlap was markedly higher in the bilateral olfactory cortex, bilateral hippocampus, bilateral caudate and putamen and right superior parietal gyrus (Figure 1a and Figure S1A for better localization of hippocampal lesions in A+D− group), suggesting a crucial role of these regions in the pathophysiology of pure apathy. The lesion subtraction plot revealed that lesion differences between the A+D− and A−D− groups were approximately 20% in the bilateral olfactory cortex, bilateral hippocampus and bilateral caudate and putamen, and 10% in the right superior parietal gyrus (Figure 1b and Figure S1B for better localization of hippocampal damage in A+D− group). These findings indicate a higher prevalence of structural damage in these regions in the A+D− group, supporting their potential involvement in apathy‐related mechanisms.

In the A−D+ group, lesion overlap was higher in the right inferior frontal gyrus, pars triangularis, bilateral olfactory cortex, right rectus, left anterior cingulate cortex, right posterior cingulate cortex, left amygdala, right calcarine fissure, left fusiform gyrus, bilateral caudate and putamen and right pallidum (Figure 1c and Figure S1C for better localization of amygdala lesions in A−D+ group), suggesting a crucial role of these regions in the pathophysiology of depression. The lesion overlap differences between the A−D+ and A−D− groups were approximately 60% in the bilateral olfactory cortex, caudate and putamen and right pallidum, and approximately 20% in the right rectus, right posterior cingulate cortex, right calcarine fissure, left amygdala and left fusiform gyrus, reflecting disproportionately high damage prevalence in these regions in A−D+ (Figure 1d and Figure S1D for better localization of amygdala damage in A−D+ group).

In the A+D+ group, lesion overlap was significantly higher in the bilateral caudate, right putamen and right insula (Figure 1e), suggesting the pivotal role of such regions in the pathophysiology of apathy and depression co‐morbidity. The lesion overlaps differences between the A+D+ and the reference group (A−D−) were approximately 10% in the bilateral caudate, right putamen and right insula, reflecting disproportionately high damage prevalence in these regions in A+D+ (Figure 1f).

## DISCUSSION

In this study, we aimed to provide novel evidence on the role of specific brain structures in mediating pure apathy, depression and their co‐occurrence in PwMS. Our findings indicate that distinct and overlapping lesion distributions in cortical and subcortical regions contribute to different clinical manifestations frequently observed in MS. These results support and extend prior literature highlighting the role of fronto‐limbic‐striatal circuits in affective and motivational symptoms (e.g. Cote et al., 2023).

Pure apathy in MS was associated with lesions in limbic structures (bilateral hippocampus, bilateral olfactory cortex) and striatal regions (bilateral caudate and putamen), as well as the right superior parietal gyrus. These regions are essential for self‐initiated movements, reward valuation, goal‐directed behaviour and action initiation (e.g. Zheng et al., 2021). Dysfunction in these areas may manifest as auto‐activation deficits, a subtype of apathy characterized by diminished volitional drive (Levy & Dubois, 2006). The involvement of the right superior parietal lobe likely reflects its role in motor planning, sensory‐driven movement strategies and attentional allocation, suggesting that impaired integration of internal and external stimuli contributes to reduced motivation (Menon & Uddin, 2010). Conversely, depression in PwMS exhibited a broader lesion distribution affecting fronto‐limbic‐subcortical circuits, aligning with the cortico–striatal–pallidal model of depression in MS. This model suggests that progressive grey matter loss in basal ganglia structures, including the globus pallidus, leads to hedonic deficits, while prefrontal atrophy contributes to maladaptive coping strategies and increased vulnerability to depressive symptoms (Stuke et al., 2016). Importantly, amygdala lesions have emerged as a key feature of depression, reflecting its central role in affective processing and mood regulation. Disrupted connectivity between the amygdala and prefrontal and limbic regions, including the cingulate cortex and inferior frontal gyrus, may underlie symptoms such as sadness and irritability (Jones et al., 2021). Lesion overlap in the right inferior frontal gyrus and fusiform gyrus suggests compromised cognitive control over negative affect and altered processing of emotionally salient stimuli, respectively, potentially contributing to emotional dysregulation in MS‐related depression (van Tol et al., 2010). Notably, the fusiform gyrus, often overlooked in depression research, is specifically implicated in emotion recognition and visual processing of salient stimuli, whose dysfunction may exacerbate mood disturbances in MS. Additionally, lesion overlap in the caudate, putamen and pallidum suggests prefrontal‐striatal loop dysfunction, a mechanism underlying mood regulation in both psychiatric and neurological disorders.

Interestingly, the A+D+ group exhibited fewer macroscopic lesions than other subgroups, particularly in the right caudate and insula. This suggests that the co‐occurrence of apathy and depression may be more closely linked to microstructural alterations and functional connectivity disruptions rather than gross structural damage (Tazza et al., 2024). The insula, a key hub of the salience network, plays a critical role in integrating cognitive, emotional and homeostatic signals to guide goal‐directed behaviour. Dysfunction in this network may impair salience attribution and motivational processes, contributing to overlapping features of apathy and depression. These findings suggest that while apathy and depression are distinct syndromes with specific lesion patterns, their co‐occurrence may be functionally driven rather than structurally, ultimately impairing goal‐directed activity and motivational behaviour.

Although our analysis focused on grey matter lesions, it is important to note that many MS lesions also affect white matter, leading to widespread network disconnection. Long‐range tracts such as the uncinate fasciculus, cingulum and superior longitudinal fasciculus are particularly vulnerable in MS and may contribute more to behavioural outcomes than lesion load alone (Cote et al., 2023; Tazza et al., 2024). This provides a plausible explanation for why the A+D+ group displayed significant symptoms despite low macroscopic lesion load. Small strategically located lesions can disrupt large‐scale network connectivity, impairing motivation and emotional regulation.

Taken together, these findings suggest that apathy and depression are partially dissociable syndromes with distinct lesion patterns but may converge functionally when network‐level connectivity is compromised. This emphasizes the need to consider distributed network dysfunction, not only focal grey matter damage, when interpreting behavioural changes in MS.

Understanding the neural underpinnings of apathy and depression in MS can inform differential diagnosis, guide targeted neuropsychological interventions and support personalized monitoring in patients with lesions in critical motivational and affective circuits. While structural lesion mapping cannot capture all facets of these complex syndromes, it provides causal insight into the brain regions most critical for specific symptoms, complementing functional and cognitive investigations.

Despite the novel findings, several methodological limitations must be acknowledged. First, MRI‐based lesion mapping may underestimate microstructural damage and does not account for neuroplasticity. Second, the lack of cognitive assessment limits the ability to explore how cognitive reserve or educational background modulates symptom expression. Third, voxel‐based lesion‐symptom mapping (VLSM), fMRI and DTI were not performed, restricting our understanding of network connectivity. Finally, the cross‐sectional design precludes conclusions about longitudinal progression. Future studies should adopt multimodal approaches integrating structural, functional and cognitive measures to clarify the complex mechanisms underlying apathy and depression in MS.

## CONCLUSION

In conclusion, our study provides novel evidence for distinct and overlapping lesion correlates of apathy and depression in MS, highlighting the importance of both focal grey matter damage and distributed network disconnections. These insights advance the understanding of motivational and affective dysfunction in MS and may ultimately support improved diagnostic precision, targeted interventions and personalized neurorehabilitation strategies.

## AUTHOR CONTRIBUTIONS

**Valentina Torchia:** Formal analysis; investigation; validation; visualization; writing – original draft; writing – review and editing; data curation. **Vera Gramigna:** Data curation; formal analysis; investigation; validation; visualization; writing – original draft; writing – review and editing. **Mariachiara Gaita:** Data curation; formal analysis; visualization; validation; investigation; writing – original draft; writing – review and editing. **Daniele Spitaleri:** Investigation. **Antonio Costanzo:** Writing – review and editing. **Liana Palermo:** Investigation; writing – review and editing. **Florindo d'Onofrio:** Investigation; writing – review and editing. **Simona Raimo:** Conceptualization; data curation; methodology; project administration; supervision; validation; visualization; writing – original draft.

## FUNDING INFORMATION

Open access funding provided by Università degli Studi Magna Graecia di Catanzaro within the CRUI‐CARE Agreement.

## CONFLICT OF INTEREST STATEMENT

The authors state that there is no conflict of interest.

## ETHICS STATEMENT

All procedures performed in studies involving human participants were in accordance with the ethical standards of the institutional and/or national research committee and with the 1964 Helsinki declaration and its later amendments or comparable ethical standards. The study was approved by the local ethics committee.

## Supporting information


Data S1.

## Data Availability

The data that support the findings of this study are available on request from the corresponding author. The data are not publicly available due to privacy or ethical restrictions.
