# Supplementary material for: Neural correlates of pure apathy in multiple sclerosis: An exploratory neuroimaging study
Source: J Neuropsychol. 2026 Mar 19;20(3):695–701. doi: 10.1111/jnp.70041 (PMC13552241; doi:10.1111/jnp.70041)
Supplement: Supplementary file 1 [file JNP-20-695-s001.docx]

***Supplementary Material***

***Participants***

A total of sixty-two PwMS, diagnosed according to the revised 2017 McDonald criteria (Thompson et al., 2018), were recruited from the Multiple Sclerosis Centre of Moscati Hospital in Avellino, Italy. All participants had a relapsing-remitting clinical course (RRMS) and had been receiving disease-modifying therapies for at least six months. The following exclusion criteria were applied: (i) contraindications to MRI; (ii) major neurocognitive disorder, as defined by Diagnostic and Statistical Manual of Mental Disorders, Fifth Edition (DSM-5; American Psychiatric Association, 2013); (iii) general intellectual decline, operationalized as a Mini-Mental State Examination (MMSE) score below 23.8, based on Italian normative data (Measso et al., 1993); (iv) severe neurological disability, indicated by an Expanded Disability Status Scale (EDSS) score > 7 (Kurtzke, 1983); (v) a history of psychiatric disorders, excluding apathy and depression; (vi) history of head trauma, other neurological diseases, or substance abuse; (vii) presence of other autoimmune diseases; and (viii) non-native Italian speakers. For each participant, demographic data (age, sex, education) and clinically relevant information (disease duration, age at onset, EDSS score, and current pharmacological treatment) were recorded. All procedures were conducted in compliance with the 1964 Declaration of Helsinki, and the study received approval from the institutional ethics committee. Written informed consent was obtained from all participants prior to participation.

***Behavioural Assessment and Group Classification***

Consistent with prior research (Raimo et al., 2016), apathy was evaluated using the self-report version of the Apathy Evaluation Scale (AES-S; Marin et al., 1991), specifically validated for use with PwMS (Raimo et al., 2014). The 18-item scale assesses multiple apathy domains (cognitive, emotional, and behavioural apathy) by means of a 4-point Likert scale, with total scores ranging from 18 to 72, where higher scores indicate greater apathy severity. Depression was assessed using the Hamilton Depression Rating Scale (HDRS-17; Hamilton, 1960), specifically validated for use with PwMS (Raimo et al., 2015), and providing total scores ranging from 0 to 54, with higher scores reflecting greater severity of depressive symptoms. Based on clinical criteria for apathy (Robert et al., 2018) and depression (DSM-5) and validated cut-off scores from previous studies, that is AES-S ≥ 36/72 for clinically significant apathy (see Raimo et al., 2014) and HDRS-17 ≥ 15/54 for clinically significant depression (see Raimo et al., 2015), we identified four distinct groups of PwMS: PwMS with neither apathy nor depression (A-D-), PwMS with pure apathy without depression (A+D-), PwMS with depression only (A-D+), and PwMS with apathy and depression (A+D+).

***Neuroimaging acquisition and lesion identification***

All PwMS had clinical 3-Tesla MRI scans. Lesion mapping was performed using MRIcron (v1.0.20190902; Rorden and Brett, 2000), following the methodology described in Boccia et al. (2020). Lesions were manually traced as volumes of interest (VOIs) on FLAIR images. The initial segmentation was performed by a trained image analyst and subsequently reviewed by an experienced neurologist (D.S.) and neuroradiologist (F.D.), both blinded to clinical data. Consensus was reached on the lesion extent for each case. FLAIR images and corresponding lesion maps were spatially normalized to a T1-weighted template from the Montreal Neurological Institute (MNI; Brett et al., 2001; Rorden et al., 2007). Normalization was performed using the unified segmentation algorithm implemented in SPM8 (Seghier, 2008) via the Clinical Toolbox for SPM (https://www.nitrc.org/projects/clinicaltbx/). Lesion localization and estimation were conducted using the Automated Anatomical Labeling (AAL) atlas (Tzourio-Mazoyer et al., 2002), allowing for the identification of commonly affected brain regions across PwMS groups (A-D-, A-D+, A+D-, A+D+). Lesion volume was quantified by calculating the total number of lesioned voxels and multiplying by the voxel size (1 mm³). Lesion distribution across PwMS subgroups (A-D-, A-D+, A+D-, A+D+) was visualized using MRIcron. Overlay maps of lesioned voxels highlighted the most frequently affected regions, applying a 10% voxel damage threshold for group comparisons. To mitigate potential biases in lesion distribution due to anatomical vulnerability (e.g., vascular density; Sarter et al., 1996; Lee and Kim, 2017), a subtraction analysis was conducted. Lesion overlap maps for the three PwMS groups exhibiting behavioural alterations (A+D-, A-D+, A+D+) were compared against the reference ‘control’ group (A-D-). Differences in lesion distribution were quantified to identify brain regions predominantly affected in specific groups.

***Statistical analysis***

Statistical analyses were performed using SPSS version 30. All results were expressed as mean ± S.D. (Standard Deviation). Clinical and demographic characteristics were analyzed by the Kruskal-Wallis analysis of variance or Chi-square (χ^2^) test. The Bonferroni post-hoc test was used for pairwise comparisons. P-values < 0.05 were considered statistically significant.

**Supplementary Figure 1. Lesion-symptom mapping for hippocampal and amygdala lesions in A+D- and A-D+ groups, respectively.** Maps of overlapping results superimposed on coronal slices of a standard brain from a single normal subject (MRIcron: ch2.betnii.gz) for **(A)** A+D-; **(C)** A-D+. Subtraction analysis results for **(B)** A+D- vs A-D-; **(D)** A-D+ vs A-D- comparisons. The colour map indicates the frequency of pathology-related lesions (maximal: red blobs).

**References**

Boccia, M., Raimo, S., Di Vita, A., Battisti, A., Matano, A., Guariglia, C., Grossi, D., & Palermo, L. (2020). Topological and hodological aspects of body representation in right brain damaged patients. Neuropsychologia, 148, 107637. https://doi.org/10.1016/j.neuropsychologia.2020.107637

Brett, M., Leff, A. P., Rorden, C., & Ashburner, J. (2001). Spatial normalization of brain images with focal lesions using cost function masking. NeuroImage, 14(2), 486–500. https://doi.org/10.1006/nimg.2001.0845

Hamilton, M. (1960). A rating scale for depression. Journal of neurology, neurosurgery, and psychiatry, 23(1), 56–62. https://doi.org/10.1136/jnnp.23.1.56

Kurtzke J. F. (1983). Rating neurologic impairment in multiple sclerosis: an expanded disability status scale (EDSS). Neurology, 33(11), 1444–1452. https://doi.org/10.1212/wnl.33.11.1444

Lee, K. B., Kim, J. S., Hong, B. Y., Sul, B., Song, S., Sung, W. J., Hwang, B. Y., & Lim, S. H. (2017). Brain lesions affecting gait recovery in stroke patients. Brain and behavior, 7(11), e00868. https://doi.org/10.1002/brb3.868

Marin, R. S., Biedrzycki, R. C., & Firinciogullari, S. (1991). Reliability and validity of the Apathy Evaluation Scale. Psychiatry research, 38(2), 143–162. https://doi.org/10.1016/0165-1781(91)90040-v

Measso, G., Cavarzeran, F., Zappalà, G., Lebowitz, B. D., Crook, T. H., Pirozzolo, F. J., Amaducci, L. A., Massari, D., & Grigoletto, F. (1993). The Mini-Mental State Examination: Normative study of an Italian random sample. Developmental Neuropsychology, 9(2), 77–85. https://doi.org/10.1080/87565649109540545

Raimo, S., Trojano, L., Spitaleri, D., Petretta, V., Grossi, D., & Santangelo, G. (2015). Psychometric properties of the Hamilton Depression Rating Scale in multiple sclerosis. Quality of life research : an international journal of quality of life aspects of treatment, care and rehabilitation, 24(8), 1973–1980. https://doi.org/10.1007/s11136-015-0940-8

Raimo, S., Trojano, L., Spitaleri, D., Petretta, V., Grossi, D., & Santangelo, G. (2014). Apathy in multiple sclerosis: a validation study of the apathy evaluation scale. Journal of the neurological sciences, 347(1-2), 295–300. https://doi.org/10.1016/j.jns.2014.10.027

Regier, D. A., Kuhl, E. A., & Kupfer, D. J. (2013). The DSM-5: Classification and criteria changes. World psychiatry: official journal of the World Psychiatric Association (WPA), 12(2), 92–98. https://doi.org/10.1002/wps.20050

Robert, P., Lanctôt, K. L., Agüera-Ortiz, L., Aalten, P., Bremond, F., Defrancesco, M., … Manera, V. (2018). Is it time to revise the diagnostic criteria for apathy in brain disorders? The 2018 international consensus group. *European Psychiatry*, *54*, 71–76. doi:10.1016/j.eurpsy.2018.07.008

Rorden, C., & Brett, M. (2000). Stereotaxic display of brain lesions. Behavioural neurology, 12(4), 191–200. https://doi.org/10.1155/2000/421719

Rorden, C., Karnath, H. O., & Bonilha, L. (2007). Improving lesion-symptom mapping. Journal of cognitive neuroscience, 19(7), 1081–1088. https://doi.org/10.1162/jocn.2007.19.7.1081

Sarter, M., Berntson, G. G., & Cacioppo, J. T. (1996). Brain imaging and cognitive neuroscience: Toward strong inference in attributing function to structure. American Psychologist, 51(1), 13–21. https://doi.org/10.1037/0003-066X.51.1.13

Seghier M. L. (2008). Laterality index in functional MRI: methodological issues. Magnetic resonance imaging, 26(5), 594–601. https://doi.org/10.1016/j.mri.2007.10.010

Thompson, A. J., Banwell, B. L., Barkhof, F., Carroll, W. M., Coetzee, T., Comi, G., Correale, J., Fazekas, F., Filippi, M., Freedman, M. S., Fujihara, K., Galetta, S. L., Hartung, H. P., Kappos, L., Lublin, F. D., Marrie, R. A., Miller, A. E., Miller, D. H., Montalban, X., Mowry, E. M., … Cohen, J. A. (2018). Diagnosis of multiple sclerosis: 2017 revisions of the McDonald criteria. The Lancet. Neurology, 17(2), 162–173. https://doi.org/10.1016/S1474-4422(17)30470-2

Tzourio-Mazoyer, N., Landeau, B., Papathanassiou, D., Crivello, F., Etard, O., Delcroix, N., Mazoyer, B., & Joliot, M. (2002). Automated anatomical labeling of activations in SPM using a macroscopic anatomical parcellation of the MNI MRI single-subject brain. NeuroImage, 15(1), 273–289. https://doi.org/10.1006/nimg.2001.0978
